# Effects of Earmuffs and Eye Masks on Propofol Sedation during Spinal Anesthesia for Orthopedic Surgery: A Randomized Controlled Trial

**DOI:** 10.3390/jcm12030899

**Published:** 2023-01-23

**Authors:** Jin-Woo Park, Sung Il Bae, Jungyul Ryu, Seung Hyun Chung, Sang-Hwan Do

**Affiliations:** 1 Department of Anesthesiology and Pain Medicine, Seoul National University Bundang Hospital, Seongnam 13620, Republic of Korea; 2 Department of Anesthesiology and Pain Medicine, College of Medicine, Seoul National University, Seoul 03080, Republic of Korea; 3 Department of Anesthesiology and Pain Medicine, Jinju Gyeongsang National University Hospital, Jinju 52727, Republic of Korea; 4 Department of Anesthesiology and Pain Medicine, Hallym University Dongtan Sacred Heart Hospital, Hwaseong 18450, Republic of Korea; 5 Department of Anesthesiology and Pain Medicine, Uijeongbu Eulji Medical Center, Eulji University, Uijeongbu 11759, Republic of Korea

**Keywords:** earmuffs, eye masks, propofol, regional anesthesia, sedation

## Abstract

Intravenous sedative drugs are commonly administered during regional anesthesia. However, reducing the excessive use of sedatives while providing adequate sedation is important from the clinical perspective, since the use of sedatives can cause considerable complications. We hypothesized that the application of earmuffs and eye masks would help reduce the sedative dose required to maintain proper sedation by blocking external stimuli. Patients who underwent orthopedic surgery under spinal anesthesia were randomly allocated to the control (no intervention) or intervention group (wearing earmuffs and eye masks). Intravenous sedation was administered using target-controlled infusion of propofol. The target concentration was controlled to maintain a Modified Observer’s Assessment of Alertness and Sedation score of 3 or 4. The primary outcome was the intraoperative propofol requirement. We also investigated the incidence of apnea, and patient satisfaction. Propofol requirement was significantly lower in the intervention group than that in the control group (2.3 (2.0–2.7) vs. 3.1 (2.7–3.4) mg·kg^−1^·h^−1^; *p* < 0.001). Intraoperative apnea occurred less frequently (*p* = 0.038) and patient satisfaction was higher (*p* = 0.002) in the intervention group compared to the control group. This study demonstrated that the use of earmuffs and eye masks during sedation was associated with lower propofol requirement and improved sedation quality.

## 1. Introduction

Intravenous sedative drugs are commonly administered during regional anesthesia to reduce patients’ anxiety and increase their comfort during surgery [1,2]. Perioperative anxiety is not restricted to simple psychological stress, but can also lead to various complications, such as increased postoperative pain and analgesic requirements and prolonged hospitalization [3,4,5].

However, sedatives should be administered cautiously because of their considerable side effects. Intravenous sedatives can cause respiratory depression and hypotension during surgery, necessitating close monitoring and prompt treatment of patients to avoid medical emergencies [6,7,8]. Thus, non-pharmacological interventions that could help reduce the dose of sedatives as well as the patient’s anxiety are important clinically.

Environmental factors should be considered when maintaining appropriate sedation during surgery [9,10,11]. Bright lighting and surgical noise in the operating room can disturb the patient’s ability to relax and interfere with proper sedation. According to previous studies, earplugs and eye masks, which were employed to reduce external noise and light stimulation, improved the patient’s quality of sleep and decreased the incidence of postoperative disorientation [9,12]. Studies have reported that playing music to patients through headphones could reduce anxiety and sedative requirements during spinal anesthesia [1,13]. However, it has not been explored whether blocking external noise and light stimulation, without any music, might help to sedate patients intraoperatively.

We hypothesized that the application of earmuffs and eye masks during spinal anesthesia would block external stimuli, help patients relax, and reduce the sedative dose required to maintain proper sedation. This study entailed a randomized controlled trial to investigate the effect of the use of earmuffs and eye masks in patients undergoing orthopedic surgeries under spinal anesthesia on the sedative dose requirements for appropriate sedation and sedation quality.

## 2. Materials and Methods

This prospective randomized control trial was approved by the Institutional Review Boards of Seoul National University Bundang Hospital (study number: B2102-664-309) and was registered at the University Hospital Medical Information Network Clinical Trials Registry (registration number: UMIN000043496). This study adhered to the CONSORT guidelines. Written informed consent was obtained from all patients before surgery. Patients were enrolled between 25 March 2021 and 4 August 2022 at Seoul National University Bundang Hospital.

### 2.1. Patients

This study enrolled adult patients aged 19–64 years, with American Society of Anesthesiologists physical status I or II, who were scheduled to undergo elective orthopedic surgeries under spinal anesthesia. Patients who underwent treatment under general or epidural anesthesia and those with a history of heart failure, respiratory failure, sleep apnea, or propofol allergy were excluded. Patients who did not want intravenous sedation, earmuffs, or eye masks during surgery were excluded. We also excluded patients undergoing procedures requiring the lateral decubitus position during surgery, owing to the impossibility of applying earmuffs in these cases. Patients who required intensive care unit admission after surgery were also excluded. The participants were randomly assigned to the control group or intervention group using a computer-generated random code (Random Allocation Software Version 1.0; University of Medical Sciences, Isfahan, Iran) in an allocation ratio of 1:1. Randomization was performed by an independent anesthesiologist who was only involved in patient assignment and measurement of the sedation score.

### 2.2. Spinal Anesthesia and Intervention

After the patient entered the operating room, standard monitoring was initiated using electrocardiography, noninvasive blood pressure, peripheral oxygen saturation, and bispectral index measurement (BIS; Medtronic, Dublin, Ireland).

Spinal anesthesia was induced with an intrathecal injection of an optimal dose of 0.5% hyperbaric bupivacaine with fentanyl 10–20 μg through a 26-G Quincke needle into the L3–4 or L4–5 interspace. After the appropriate level of sensory blockade and hemodynamic stability were confirmed, earmuffs (Optime 105, 3M, Saint Paul, MI, USA) and eye masks (Dreamweaver-Contoured Sleep Mask, MACK’S, Michigan, IN, USA) were placed over the ears and eyes, respectively, of the patients in the intervention group. Earmuffs and eye masks were not used in the control group. The eye masks were discarded after single use and the earplugs were cleaned with an alcohol-containing cleaner.

Intravenous sedation was provided after intervention. Target-controlled infusion (TCI) of propofol was performed using an Orchestra infusion pump system (Fresenius vial, Berzins, France); the initial target effect-site concentration was set to 1.2 μg·mL^−1^. During sedation, the Modified Observer’s Assessment of Alertness and Sedation (MOAA/S) was assessed every 10 min by an independent anesthesiologist who was responsible for patient allocation. For patients in the intervention group, the earmuffs were lifted slightly from the left ear for sedation assessment. The definition of the MOAA/S score was as follows: (5) responds readily to name spoken in normal tone, (4) lethargic response to name spoken in normal tone, (3) responds only after the name is called loudly and/or repeatedly, (2) responds only after mild prodding or shaking, and (1) does not respond to mild prodding or shaking. The target sedation score was 3 or 4, and the effect-site concentration was adjusted according to the MOAA/S score every 10 min as follows: if the MOAA/S was 5, the target concentration was increased by 0.2 μg·mL^−1^; if it was 3 or 4, the concentration was maintained as the same; if the MOAA/S score was 2 or less, the target effect-site concentration was decreased by 0.2 μg·mL^−1^. During surgery, 10–20 µg of phenylephrine was administered intravenously when the mean blood pressure (MBP) decreased by more than 20% from baseline or fell below 60 mmHg. Atropine 0.5 mg was administered when the heart rate fell below 40 beats/min.

### 2.3. Study Outcomes

The primary outcome was the total intraoperative requirement of propofol, which was defined as the total dose of propofol · patient’s body weight^−1^ · administration time^−1^ (mg·kg^−1^·h^−1^). The MBP, heart rate, BIS score, and target effect-site concentration of propofol were recorded every 10 min during surgery. The time taken to reach a BIS value of 80 or less from the start of propofol administration was also measured and termed as the induction time. The incidence of apnea due to sedative administration and inotropic requirements during surgery were also recorded. An independent blinded researcher asked patients to estimate their satisfaction with intraoperative sedation using a numerical rating scale (score: 0, very dissatisfied, to 10, very satisfied) 24 h after surgery. The incidence of postoperative nausea and vomiting during the first 24 h was also investigated.

### 2.4. Statistical Analysis

Continuous data were expressed as median (interquartile range) and categorical variables were presented as numbers (percentages). Continuous outcomes between the control and intervention groups were compared using the Mann–Whitney U test. The chi-squared or Fisher exact test was used to analyze the categorical outcomes. SPSS version 21.0 (SPSS Inc., IBM, Chicago, IL, USA) was used for all statistical analyses. Statistical significance was defined as a two-sided *p*-value of <0.05.

### 2.5. Sample Size Calculation

In a pilot study of 20 patients (10 in each group) undergoing orthopedic surgery under spinal anesthesia, the mean (standard deviation) propofol requirement was 3.3 (0.8) and 2.8 (0.7) mg·kg^−1^·h^−1^ for the control and intervention groups, respectively. A power analysis was performed with G*Power 3.1.2 (Heinrich-Heine University, Düsseldorf, Germany) on the basis of the results of the pilot study. Our calculation showed that a sample size of 40 patients per group would ensure a statistical power of 80%, after accounting for a risk of 0.05 for type I errors in two-tailed analysis, and a 10% dropout rate.

## 3. Results

**Figure 1:**
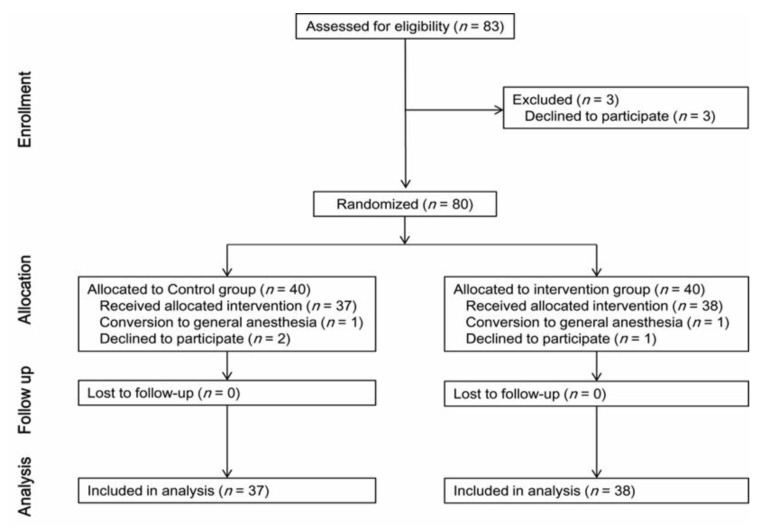
Flow chart of patient selection.

A total of 3 of the 83 eligible patients declined to participate in the study, and 80 patients were randomly allocated to the control (40 patients) or intervention group (40 patients). The anesthetic method was changed to general anesthesia for one patient in each group. Two patients in the control group and one patient in the intervention group declined to participate in the study before spinal anesthesia. Therefore, 75 patients completed the study (37 in the control group and 38 in the intervention group; Figure 1).

The patient characteristics did not differ between the two groups (Table 1). Surgical data, including surgery/sedation time, spinal anesthetics, and type of surgery, did not differ between the groups (Table 2).

The intraoperative propofol requirement for controlled sedation was significantly lower in the intervention group than that in the control group (2.3 (2.0–2.7) vs. 3.1 (2.7–3.4) mg·kg^−1^·h^−1^; *p* < 0.001; Table 3). The mean effect-site concentration of propofol was also lower in the intervention group than that in the control group during the surgery (0.9 (0.8–1.1) vs. 1.2 (1.1–1.4) mcg·mL^−1^; *p* < 0.001). The mean BIS did not significantly differ between the groups (*p* = 0.478). The induction time was shorter in the intervention group than that in the control group (190.0 (120.0–321.0) vs. 280.0 (180.0–452.0) s, *p* = 0.006). The incidence of intraoperative apnea was significantly lower in the intervention group (3 vs. 11, *p* = 0.038). The incidence of postoperative nausea and vomiting was comparable between the two groups (*p* = 0.190). Patient satisfaction with intraoperative sedation was significantly higher in the intervention group than that in the control group (10.0 (9.0–10.0) vs. 8.0 (8.0–10.0), *p* = 0.002).

The basal MBP and heart rate were comparable between the control and intervention groups (*p* = 0.379 and 0.461, respectively; Table 4). However, the intraoperative MBP was significantly higher in the intervention group (77.3 (72.9–96.1) vs. 75.1 (69.5–78.9) mmHg; *p* = 0.036) than that in the non-intervention group. The mean heart rate did not differ significantly between the two groups during surgery (*p* = 0.546), and neither did the intraoperative inotropic requirement (*p* = 0.563).

The subgroup analysis of propofol use was also performed for cases with high noise and low noise levels (Table 4). During orthopedic surgery with high noise level as well as low noise, propofol dose was significantly lower in the intervention group compared with that in the control group (*p* < 0.001 for both levels). Likewise, mean propofol target concentration during surgery was also lower in the intervention group than that in the control group for both noise levels (*p* = 0.023 and 0.001 for high noise level surgery and low noise, respectively).

## 4. Discussion

This randomized controlled study confirmed that the application of earmuffs and eye masks to block external stimuli significantly decreased the propofol dose required for controlled sedation during orthopedic surgery under spinal anesthesia. The intervention group also showed a lower mean effect-site concentration of propofol, shorter induction time, lower frequency of apnea, and greater patient satisfaction compared to the control group. This was the first clinical trial to investigate the effect of earmuffs and eye masks on the sedative dose and sedation quality during spinal anesthesia.

Excessive propofol use for sedation during spinal anesthesia could be detrimental for the patient. Deep sedation using propofol infusion is reportedly associated with greater postoperative delirium and even mortality [14,15,16]. Therefore, the administration of appropriate sedative doses to maintain proper sedation are vital. In this study, the effect-site concentration of propofol was lower in the intervention group than that in the control group, albeit the BIS values were similar in both groups; thus, blocking external stimuli seemed to aid in the relaxation and sedation of patients intraoperatively. Moreover, the induction time was also significantly shorter in the intervention group, even though the initial target effect-site concentration of propofol was the same in both groups. A previous study reported that blocking noise by packing the ears significantly reduced the BIS score during propofol-induced sedation in noisy environments, which is consistent with our results [10].

The intervention in our protocol reduced the incidence of intraoperative apnea, which may be attributed to the significantly decreased propofol dose in the intervention group. Intraoperative apnea is one of the most common and critical complications of propofol administration [17,18,19]. It is fundamentally critical that anesthesiologists maintain spontaneous ventilation during intravenous sedation, and avoid hypercapnia or hypoxemia, because sedative agents inevitably depress respiration [20]. Even respiratory arrest cases due to intravenous sedation have been reported [21]; the role of anesthesiologists to direct intravenous sedation outside the operating room as well as intraoperatively is becoming increasingly important [22,23].

There was also a significant difference in the intraoperative MBP between the two groups, however, the hemodynamic difference did not seem clinically significant. Since the basal MBP was comparable between the two groups before the initiation of sedation, the difference of MBP may be attributed to the difference in the propofol dose between them. Along with respiratory depression, hypotension is one of well-known side effects of propofol infusion [24,25]. However, the inotropic requirement or intraoperative heart rate did not differ between the groups.

In this study, both the BIS and MOAA/S were assessed during TCI of propofol. TCI has been utilized for propofol sedation in patients with spinal anesthesia at our institution, owing to its superiority over the manually controlled method with respect to hemodynamic stability and convenience of management [1,26]. MOAA/S is a more sensitive sedation monitoring method than BIS in patients under spinal anesthesia [27]. Therefore, in our protocol, the target concentration of TCI propofol was regulated according to the MOAA/S score every 10 min. However, BIS is also known to have a good correlation with the sedation depth during propofol anesthesia [28,29,30]; we defined the induction time based on BIS and compared the BIS profile between the two groups.

A previous study found that (listening to) music significantly decreased the induction time and propofol dose required for sedation during regional anesthesia [1]. However, this approach would require greater attention to detail and preparation, such as determining the patient’s music preference, volume level, and arrangement of audio devices, compared to our intervention. Earmuffs and eye masks can be applied simply and easily in various clinical settings. Furthermore, noise blockade reportedly reduces BIS more effectively than listening to music in noisy environments [10].

Research on the effects of earplugs and eye masks during sleep has shown that they significantly improve sleep quality [9,12]. However, controlled clinical data on the effects of active blockade of external stimuli on sedation quality are insufficient. In our study, patient satisfaction with intraoperative sedation was also significantly higher in the intervention group than that in the control group. Some patients in the control group complained of noise or inappropriate conversations among the medical staff, which could be experienced by patients when intraoperative sedation was insufficient, especially at the beginning of propofol administration. Earmuffs and eye masks can also interrupt these unnecessary stimuli.

To check the effects of earmuffs and eye masks at different intraoperative noise levels, we performed the subgroup analysis of propofol use in cases with high noise and low noise levels. The analysis confirmed that, during surgery with high noise level as well as low noise, propofol dose and propofol target concentration were significantly lower in the intervention group than those in the control group. It seems that earmuffs effectively blocked external noise stimuli, regardless of the level of noise during surgery, causing a clinically significant improvement in sedation quality.

This study has some limitations. First, the attending anesthesiologists and patients were not blinded to the allocated intervention. However, each procedure or assessment was performed precisely, according to the study protocol. Patient allocation, MOAA/S monitoring, and propofol concentration adjustment were performed by an independent anesthesiologist who did not participate in other anesthetic procedures. Furthermore, postoperative outcomes were measured by one blinded researcher. Second, we utilized a numerical rating scale to estimate patient satisfaction. Other instruments such as the Evaluation du Vécu de l’Anesthésie LocoRégionale survey might be more robust [31,32,33]. However, we needed to focus on intraoperative sedation rather than on the entire anesthesia process; a blinded researcher tried to help patients to freely self-assess their satisfaction about the quality of sedation. Third, we enrolled patients who agreed to wearing earmuffs and eye masks, which could contain a selection bias, as only patients who were expecting positive effects were included in the protocol. However, the number of patients who refused the intervention was relatively small compared to the total number of screened patients. Moreover, it should be a clinically important finding that the intervention in patients who did not have an objection to wearing earmuffs and eye masks significantly reduced propofol dose and improve sedation quality. Fourth, the generalizability of our results is limited because this study was conducted at a single medical center. Multicenter data are required to confirm the generalizability of these results.

In conclusion, this prospective study demonstrated that the application of earmuffs and eye masks during elective orthopedic surgery with propofol sedation under spinal anesthesia was helpful in reducing propofol requirement, which also reduced the pharmacological side effects and enhanced patient satisfaction. Further studies will be needed to confirm the effects of the intervention on other sedatives and in different clinical environments.

## Figures and Tables

**Table 1:** Baseline characteristics before and after propensity score matching.

	Control Group (*n* = 37)	Intervention Group (*n* = 38)	*p*-Value
Age, years	52.0 (39.0–60.0)	54.5 (41.0–61.8)	0.248
Male, *n*	16	18	0.720
Weight, kg	68.0 (64.0–85.0)	74.1 (63.9–83.4)	0.618
Height, cm	163.0 (156.0–168.0)	162.5 (155.1–170.6)	0.931
ASA grade, I/II	17/20	18/20	0.902

Values are presented as median (IQR) or number. ASA, American Society of Anesthesiologists.

**Table 2:** Operational data.

	Control Group (*n* = 37)	Intervention Group (*n* = 38)	*p*-Value
Surgery time, min	125.0 (105.0–165.0)	115.0 (106.3–173.8)	0.706
Sedation time, min	86.0 (70.0–120.0)	80.0 (60.0–108.8)	0.257
Intrathecal bupivacaine dose, mg	13.0 (11.3–13.0)	12.5 (12.0–13.8)	0.494
Intrathecal fentanyl dose, mcg	20.0 (15.0–20.0)	15.0 (10.0–20.0)	0.177
Type of surgery, *n*			
TKRA	6	8	
Knee arthroscopic surgery	15	17	0.264
Ankle arthroscopic surgery	5	7	
ORIF	6	3	
Others ^1^	5	3	

Values are presented as median (IQR) or number. TKRA, total knee replacement arthroplasty; ORIF, open reduction and internal fixation. ^1^ Others group consisted of a variety of procedures with a small number of cases, which included wound debridement, osteotomy, mass excision, and total ankle arthroplasty.

**Table 3:** Anesthesia and complication data.

	Control Group (*n* = 37)	Intervention Group (*n* = 38)	Odds Ratio (95% CI)	*p*-Value
Propofol dose, mg·kg^−1^·h^−1^	3.1 (2.7–3.4)	2.3 (2.0–2.7)		<0.001
Mean propofol target concentration, μg·mL^−1^	1.2 (1.1–1.4)	0.9 (0.8–1.1)		<0.001
Mean BIS	76.3 (74.2–79.8)	75.9 (73.5–79.0)		0.478
Induction time, s	280.0 (180.0–452.0)	190.0 (120.0–321.0)		0.006
Basal MBP, mmHg	81.0 (74.0–89.0)	79.5 (75.0–94.9)		0.379
Mean MBP, mmHg	75.1 (69.5–78.9)	77.3 (72.9–86.1)		0.036
Basal heart rate, beat/min	71.0 (59.0–76.0)	66.0 (60.0–77.0)		0.461
Mean heart rate, beat/min	61.9 (58.2–67.2)	63.4 (56.0–71.9)		0.546
Inotropic requirement, *n*	17	20	1.3 (0.5–3.2)	0.563
Apnea incidence, *n*	11	3	0.3 (0.1–1.0)	0.038
PONV, *n*	8	4	0.4 (0.1–1.6)	0.190
Patient satisfaction	8.0 (8.0–10.0)	10.0 (9.0–10.0)		0.002

Values are presented as median (IQR) or number. BIS, bispectral index; MBP, mean blood pressure; PONV, postoperative nausea and vomiting.

**Table 4:** Intraoperative propofol dose and mean propofol target concentration among surgeries with high noise or low noise level.

	Control Group	Intervention Group	*p*-Value
High noise level ^1^	*n* = 12	*n* = 11	
Propofol dose, mg·kg^−1^·h^−1^	3.3 (3.1–3.5)	2.4 (2.2–2.6)	<0.001
Mean propofol target concentration, μg·mL^−1^	1.2 (1.2–1.2)	0.8 (0.8–1.0)	0.023
Low noise level ^2^	*n* = 20	*n* = 24	
Propofol dose, mg·kg^−1^·h^−1^	3.0 (2.6–3.4)	2.2 (1.9–2.6)	<0.001
Mean propofol target concentration, μg·mL^−1^	1.0 (0.8–1.2)	0.8 (0.6–1.0)	0.001

Values are presented as median (IQR). ^1^ High noise level subgroup included total knee replacement arthroplasty and open reduction/internal fixation. ^2^ Low noise level subgroup included knee and ankle arthroscopic surgeries.

## Data Availability

The datasets used and/or analyzed during the current study are available from the corresponding author on reasonable request.

## References

[1] Zhang X.W., Fan Y., Manyande A., Tian Y.K., Yin P. (2005). Effects of music on target-controlled infusion of propofol requirements during combined spinal-epidural anaesthesia. Anaesthesia.

[2] Yoo S.W., Ki M.J., Kim D., Oh Y.J., Lee J. (2021). The effect of an eye mask on midazolam requirement for sedation during spinal anesthesia: A randomized controlled trial. BMC Anesthesiol..

[3] Jawaid M., Mushtaq A., Mukhtar S., Khan Z. (2007). Preoperative anxiety before elective surgery. Neurosciences.

[4] Nigussie S., Belachew T., Wolancho W. (2014). Predictors of preoperative anxiety among surgical patients in Jimma University Specialized Teaching Hospital, South Western Ethiopia. BMC Surg..

[5] Kehlet H., Jensen T.S., Woolf C.J. (2006). Persistent postsurgical pain: Risk factors and prevention. Lancet.

[6] Lee M.H., Yang K.H., Lee C.S., Lee H.S., Moon S.Y., Hwang S.I., Song J.H. (2011). The effect-site concentration of propofol producing respiratory depression during spinal anesthesia. Korean J. Anesthesiol..

[7] Conrad B., Larsen R., Rathgeber J., Lange H., Stuber H., Crozier T. (1990). Propofol infusion for sedation in regional anesthesia. A comparison with midazolam. Anasth Intensiv. Notf..

[8] Frank L.R., Strote J., Hauff S.R., Bigelow S.K., Fay K. (2006). Propofol by infusion protocol for ED procedural sedation. Am. J. Emerg. Med..

[9] Hu R.F., Jiang X.Y., Zeng Y.M., Chen X.Y., Zhang Y.H. (2010). Effects of earplugs and eye masks on nocturnal sleep, melatonin and cortisol in a simulated intensive care unit environment. Crit. Care.

[10] Kang J.G., Lee J.J., Kim D.M., Kim J.A., Kim C.S., Hahm T.S., Lee B.D. (2008). Blocking noise but not music lowers bispectral index scores during sedation in noisy operating rooms. J. Clin. Anesth..

[11] Al-Samsam R.H., Cullen P. (2005). Sleep and adverse environmental factors in sedated mechanically ventilated pediatric intensive care patients. Pediatr. Crit. Care Med..

[12] Le Guen M., Nicolas-Robin A., Lebard C., Arnulf I., Langeron O. (2014). Earplugs and eye masks vs routine care prevent sleep impairment in post-anaesthesia care unit: A randomized study. Br. J. Anaesth..

[13] Lepage C., Drolet P., Girard M., Grenier Y., DeGagne R. (2001). Music decreases sedative requirements during spinal anesthesia. Anesth Analg..

[14] Brown C.H.T., Azman A.S., Gottschalk A., Mears S.C., Sieber F.E. (2014). Sedation depth during spinal anesthesia and survival in elderly patients undergoing hip fracture repair. Anesth Analg..

[15] Sieber F.E., Zakriya K.J., Gottschalk A., Blute M.R., Lee H.B., Rosenberg P.B., Mears S.C. (2010). Sedation depth during spinal anesthesia and the development of postoperative delirium in elderly patients undergoing hip fracture repair. Mayo Clin. Proc..

[16] Kwon M.Y., Lee S.Y., Kim T.Y., Kim D.K., Lee K.M., Woo N.S., Chang Y.J., Lee M.A. (2012). Spectral entropy for assessing the depth of propofol sedation. Korean J. Anesthesiol..

[17] Aikawa M., Uesato M., Urahama R., Hayano K., Kunii R., Kawasaki Y., Isono S., Matsubara H. (2020). Predictor of respiratory disturbances during gastric endoscopic submucosal dissection under deep sedation. World J. Gastrointest. Endosc..

[18] Calderwood A.H., Chapman F.J., Cohen J., Cohen L.B., Collins J., Day L.W., Early D.S. (2014). Guidelines for safety in the gastrointestinal endoscopy unit. Gastrointest. Endosc..

[19] Sasaki T., Tanabe S., Azuma M., Sato A., Naruke A., Ishido K., Katada C., Higuchi K., Koizumi W. (2012). Propofol sedation with bispectral index monitoring is useful for endoscopic submucosal dissection: A randomized prospective phase II clinical trial. Endoscopy.

[20] Shin H.J., Kim E.Y., Hwang J.W., Do S.H., Na H.S. (2018). Comparison of upper airway patency in patients with mild obstructive sleep apnea during dexmedetomidine or propofol sedation: A prospective, randomized, controlled trial. BMC Anesthesiol..

[21] Kim J.H., Byun S., Choi Y.J., Kwon H.J., Jung K., Kim S.E., Park M.I., Moon W., Park S.J. (2020). Efficacy and Safety of Etomidate in Comparison with Propofol or Midazolam as Sedative for Upper Gastrointestinal Endoscopy. Clin. Endosc..

[22] Buxbaum J., Roth N., Motamedi N., Lee T., Leonor P., Salem M., Gibbs D., Vargo J. (2017). Anesthetist-Directed Sedation Favors Success of Advanced Endoscopic Procedures. Am. J. Gastroenterol..

[23] Goudra B.G., Singh P.M., Gouda G., Borle A., Gouda D., Dravida A., Chandrashakhara V. (2015). Safety of Non-anesthesia Provider-Administered Propofol (NAAP) Sedation in Advanced Gastrointestinal Endoscopic Procedures: Comparative Meta-Analysis of Pooled Results. Dig. Dis. Sci..

[24] Sneyd J.R., Absalom A.R., Barends C.R.M., Jones J.B. (2022). Hypotension during propofol sedation for colonoscopy: A retrospective exploratory analysis and meta-analysis. Br. J. Anaesth..

[25] Mannion S. (2007). Sedation, spinal anesthesia and older patients. J. Postgrad. Med..

[26] Passot S., Servin F., Allary R., Pascal J., Prades J.M., Auboyer C., Molliex S. (2002). Target-controlled versus manually-controlled infusion of propofol for direct laryngoscopy and bronchoscopy. Anesth Analg..

[27] Pollock J.E., Neal J.M., Liu S.S., Burkhead D., Polissar N. (2000). Sedation during spinal anesthesia. Anesthesiology.

[28] Struys M.M.R.F., Vereecke H., Moerman A., Jensen E.W., Verhaeghen D., De Neve N., Dumortier F.J.E., Mortier E.P. (2003). Ability of the Bispectral Index, Autoregressive Modelling with Exogenous Input-derived Auditory Evoked Potentials, and Predicted Propofol Concentrations to Measure Patient Responsiveness during Anesthesia with Propofol and Remifentanil. Anesthesiology.

[29] Liu J., Singh H., White P.F. (1997). Electroencephalographic bispectral index correlates with intraoperative recall and depth of propofol-induced sedation. Anesth Analg..

[30] Singh H. (1999). Bispectral index (BIS) monitoring during propofol-induced sedation and anaesthesia. Eur. J. Anaesthesiol..

[31] Maurice-Szamburski A., Bruder N., Loundou A., Capdevila X., Auquier P. (2013). Development and validation of a perioperative satisfaction questionnaire in regional anesthesia. Anesthesiology.

[32] Wu C.L., Naqibuddin M., Fleisher L.A. (2001). Measurement of patient satisfaction as an outcome of regional anesthesia and analgesia: A systematic review. Reg. Anesth. Pain Med..

[33] Wu C.L., Fleisher L.A. (2000). Outcomes research in regional anesthesia and analgesia. Anesth Analg..

